# Investigating the relationship between psoriasis and venous thromboembolism using systematic review, meta-analysis and Mendelian randomization study

**DOI:** 10.1097/MD.0000000000044311

**Published:** 2025-09-05

**Authors:** Mingzhu Yang, Haiwei Zhang, Jiao Ma, Chen Xing, Hao Xu

**Affiliations:** a Department of Ultrasonic Medicine, The People’s Hospital of Danyang, Affiliated Danyang Hospital of Nantong University, Jiangsu Province, China; b Department of Vascular Surgery, The People’s Hospital of Danyang, Affiliated Danyang Hospital of Nantong University, Jiangsu Province, China; c Department of Medical Imaging, The People’s Hospital of Danyang, Affiliated Danyang Hospital of Nantong University, Jiangsu Province, China; d Department of Respiratory and Critical Care Medicine, The People’s Hospital of Danyang, Affiliated Danyang Hospital of Nantong University, Jiangsu Province, China.

**Keywords:** deep vein thrombosis, Mendelian randomization, meta-analysis, psoriasis, pulmonary embolism, venous thromboembolism

## Abstract

**Background:**

Numerous studies have investigated the correlation between psoriasis and venous thromboembolism (VTE). However, the findings have not been entirely conclusive. The objective of this study was to assess the association between psoriasis and the risk of VTE by conducting a systematic review and meta-analysis, complemented by Mendelian randomization (MR) analysis to evaluate potential causality.

**Methods:**

In our study, we utilize meta-analysis and MR to delve into the potential relationship between psoriasis and VTE. A comprehensive literature search was conducted across PubMed, Web of Science, and Embase. Different measures of association reported in the original studies – including relative risks (RRs), hazard ratios (HRs), standardized incidence ratios (SIRs), and odds ratios (ORs) – were converted to ORs for consistency using validated methods. MR was subsequently utilized to evaluate the causal impact of psoriasis on the occurrence of VTE.

**Results:**

In the primary analysis, all thromboembolic events – including pulmonary embolism (PE), deep vein thrombosis (DVT), or both – were classified as overall VTE. Meta-analysis demonstrated a higher odd of overall VTE in patients with psoriasis (OR: 1.23, 95% confidence interval (CI): 1.03–1.46). Subgroup analyses revealed that psoriasis was associated with an increased incidence of VTE in European (OR: 1.51, 95% CI: 1.34–1.70) and Asian (OR: 2.02, 95% CI: 1.42–2.88) populations, while no significant association in North American studies (OR: 0.98, 95% CI: 0.88–1.10). The RR group demonstrated a substantial increase in VTE risk among psoriasis patients (RR: 1.30, 95% CI: 1.01–1.68), as did the SIR group (SIR: 1.40, 95% CI: 1.31–1.50) and OR group (OR: 0.95, 95% CI: 0.90–0.99). Conversely, the HR group (HR: 1.19, 95% CI: 0.94–1.51), did not show a significant association. VTE type subgroup analyses were subsequently conducted to separately assess the odds of VTE, PE, and DVT as distinct outcomes. Psoriasis was found to increase the incidence of VTE-only (OR: 1.27, 95% CI: 1.03–1.56), but no significant association was observed with PE (OR: 1.13, 95% CI: 0.70–1.83) and DVT (OR: 0.85, 95% CI: 0.65–1.11). MR suggested that genetically predicted psoriasis is not associated with an increased odd of VTE (inverse-variance weighted OR: 1.000, 95% CI: 0.999–1.001, *P* = .639).

**Conclusion:**

While the meta-analysis revealed a significantly increased odds of VTE in patients with psoriasis, the MR analysis did not support a causal relationship. These findings suggest a potential observational association without confirming causality.

## 1. Introduction

Psoriasis is a common chronic inflammatory skin disease, with a global overall prevalence of approximately 2%, but there are significant regional variations.^[1–3]^ Immune system dysregulation and abnormal inflammatory responses play a crucial role in the development of psoriasis, which can affect multiple organs and systems in the body. The most common comorbidity is psoriatic arthritis,^[4]^ and it is also associated with inflammatory bowel disease,^[5]^ renal diseases,^[6]^ cardiovascular diseases^[7]^, and malignancies,^[8]^ significantly impacting the quality of life and psychological well-being of affected individuals.

Venous thromboembolism (VTE) is a severe blood circulation disorder that encompasses deep vein thrombosis (DVT) and pulmonary embolism (PE). It is estimated that millions of people worldwide are affected by VTE each year, with many cases resulting in severe complications, even fatalities. The 3 fundamental elements contributing to the occurrence of VTE are vascular endothelial injury, a hypercoagulable state of the blood and venous stasis.^[9]^ Traditional risk factors for VTE include prolonged immobilization, major surgeries, advanced age, malignancy, pregnancy, and prior history of VTE.^[10,11]^ In recent years, chronic inflammatory diseases have also been recognized as potential contributors to VTE risk,^[12]^ further highlighting the importance of systemic inflammation in thrombogenesis.

Based on the similarities in their underlying pathophysiology, recent studies have proposed a potential association between psoriasis and VTE. Several studies have reported a higher risk of VTE in patients with psoriasis,^[13–19]^ and consistent results have been observed in concurrent meta-analyses.^[20–23]^ However, some recent studies have failed to observe a significant correlation.^[24–26]^ These inconsistencies may arise from differences in study design, population characteristics, psoriasis severity, and outcome definitions, which necessitate a more rigorous and integrative evaluation. To address these discrepancies and provide a more robust assessment, we conducted a comprehensive meta-analysis and Mendelian randomization (MR) study to evaluate the potential causal association between psoriasis and VTE. By combining data from observational studies and genetic instruments, this dual-method approach enhances causal inference and mitigates residual confounding. This study aims to provide a more accurate estimate of the association and offer more reliable evidence to inform future disease prevention and clinical decision-making.

## 2. Methods

This study is a meta-analysis and MR analysis based on previously published data. All data used were aggregated and publicly available, and no individual patient data were involved. Therefore, ethical approval and informed consent were not required. To comprehensively investigate the relationship between psoriasis and VTE, we employed a 2-pronged approach combining traditional meta-analysis and MR. The meta-analysis aimed to synthesize existing observational evidence from published studies, quantifying the association between psoriasis and VTE using pooled effect estimates. The MR analysis was subsequently performed to assess potential causality by leveraging genetic variants associated with psoriasis as instrumental variables (IVs). Below, we describe the methodologies of the meta-analysis and MR analyses in detail.

## 3. Meta-analysis

### 3.1. Data source and search strategy

We conducted a comprehensive search of relevant published studies in databases including PubMed, Embase, and Web of Science. The search was performed from the inception of these databases up until June 13, 2024. The search terms used for the study included “psoriasis” in combination with “venous thromboembolism,” “VTE,” “deep vein thrombosis,” “DVT,” “pulmonary embolism.” Furthermore, the reference lists of selected articles were manually screened to identify any additional relevant studies.

### 3.2. Inclusion and exclusion criteria

We included observational studies (both cohort and case-control designs) that reported associations between psoriasis and VTE (including DVT and PE). Most included studies were based on retrospective administrative databases with matched case and control groups. The studies provided measures of association such as odds ratios (ORs), relative risks (RRs), hazard ratios (HRs), or standardized incidence ratios (SIRs), along with their corresponding 95% confidence intervals (CIs). We excluded reviews, letters, meta-analyses, meeting abstracts, nonclinical reports, and studies with insufficient data.

### 3.3. Data extraction

Data extraction was independently performed by Mingzhu Yang and Haiwei Zhang from the selected studies. In cases of discrepancies, a third researcher, Jiao Ma, was consulted to reach a consensus. The extracted data included the first author’s name, publication year, data source, study region, study design, duration of follow-up, traits, sample size, outcome measures and their corresponding CIs.

### 3.4. Quality assessment

The quality of the studies included in our analysis was independently assessed by Mingzhu Yang and Haiwei Zhang using the Newcastle–Ottawa scale (NOS). The NOS assesses studies based on 3 components: selection (0–4 points), comparability (0–2 points), and outcome measures (0–3 points). Studies that scored 6 or higher on the NOS were deemed to be of high quality. Please refer to Table 1 and Figure S1, Supplemental Digital Content, https://links.lww.com/MD/P852 for more details on the quality assessment.

**Table 1:** Main characteristics of all the studies included in the meta-analysis.

Author	Year	Data source	Study region	Type	Study design	Follow-up	Trait	Case	Control	HR/RR/SIR/OR (95% CI)	NOS
Ahlehoff^[13]^	2011	National Prescription Registry, National Causes of Death Register, Central Population Register	Danmark	Pro	Cohort	1997–2006	Mild psoriasis and VTE	35,138	41,26,075	RR 1.35 (1.21–1.49)	9
							Severe psoriasis and VTE	3526		RR 2.06 (1.63–2.61)	
Sreeram^[14]^	2011	English National Hospital Episode Statistics	England	Retro	Cohort	1963–1998	ORLS1 psoriasis and VTE	3910	36,21,957	RR 1.62 (1.32–1.98)	7
						1999–2008	ORLS2 psoriasis and VTE	3365		RR 1.65 (1.23–2.17)	
						1999–2008	England psoriasis and VTE	85,358		RR 1.66 (1.57–175)	
Lutsey^[15]^	2012	Lowa Women’s Health Study	Iowa, USA	Pro	Cohort	1991–2004	Women with psoriasis and VTE	859	37,749	HR 1.39 (1.00–1.93)	8
Zoller^[16]^	2012	MigMed2	Sweden	Retro	Cohort	1964–2008	Psoriasis and PE	25,869	5,09,669	SIR 1.40 (1.31–1.50)	9
Chung^[17]^	2017	Taiwan National Health Insurance Research Database	Taiwan, China	Retro	Cohort	2000–2010	Psoriasis and VTE	8945	8945	HR 2.02 (1.42–2.88)	7
Ogdie^[18]^	2018	The Health Improvement Network	USA	Retro	Cohort	1994–2014	Psoriasis and VTE	1,94,288	12,25,571	HR 1.08 (1.03–1.12)	9
Mohammed^[24]^	2021	National Inpatient Sample	USA	Retro	Case-control	2002–2012	Psoriasis and VTE	1,85,763	7,21,18,738	OR 0.95 (0.90–0.99)	8
Juliana^[25]^	2021	IBM MarketScan Commercial and Medicare Supplemental Database	USA	Retro	Cohort	2014–2018	Psoriasis and DVT	N/A	1,82,431	IRR 0.85 (0.65–1.11)	8
							Psoriasis and PE	N/A		IRR 0.86 (0.61–1.22)	
Maria^[26]^	2021	Provincial Hospitalization Database	USA	Retro	Cohort	2004–2019	Psoriasis and VTE	82,887	82,887	HR 0.86 (0.75–0.99)	9

CI = confidence interval, DVT = deep vein thrombosis, HR = hazard ratio, IRR = incidence rate ratio, N/A = not applicable, NOS = Newcastle–Ottawa scale, OR = odds ratio, ORLS = Oxford Record Linkage Study, PE = pulmonary embolism, Pro = prospective, Retro = retrospective, RR = relative risk, SIR = standardized incidence ratio, VTE = venous thromboembolism.

### 3.5. Statistical analysis

The association between psoriasis and VTE was evaluated by calculating effect estimates including ORs, RRs, HRs, and SIRs their corresponding 95% CIs. To ensure comparability across studies, effect estimates including HRs, RRs, and SIRs were converted to ORs prior to the meta-analysis. HRs and RRs were converted using the method proposed by Zhang and Tyler,^[27,28]^ assuming a constant incidence in the reference group. SIRs were interpreted as approximations of ORs, assuming low incidence of VTE events. Heterogeneity among the included studies was assessed using Cochran’s *Q* test and the Higgins *I*-squared test. In cases where significant heterogeneity was present (indicated by an *I*^2^ > 50% or a *P*-value < .05), a random-effects model (DerSimonian–Laird method) was employed. Subgroup analyses were conducted based on geographic region (e.g., Europe, Asia, North America) and type of effect measure (e.g., OR, HR, SIR, RR). To account for variations in outcome definitions across studies, all thromboembolic events – including VTE, PE, and DVT – were initially classified as overall VTE in the primary analysis. Furthermore, additional subgroup analyses were performed to separately assess studies that specifically reported VTE, PE, or DVT as distinct outcomes. Meta-regression was then conducted to explore the potential impact of these study-level variables on between-study heterogeneity. To test the robustness of the pooled estimates, sensitivity analyses were carried out by sequentially excluding individual studies. Publication bias was assessed using funnel plots and Egger’s test for outcomes with at least 10 included studies, with statistical significance set at *P* < .05. All statistical analyses were performed using STATA software version 12.0.

## 4. Mendelian randomization

### 4.1. Study design

The MR method relies on 3 fundamental hypotheses: Single nucleotide polymorphisms (SNPs) exhibit a robust association with psoriasis. SNPs are not influenced by known confounding factors. The influence of SNPs on VTE/DVT/PE occurs solely through their impact on psoriasis.

### 4.2. Data source

The analysis utilized publicly available summary-level data from genome-wide association studies (GWAS) that focused on relevant traits, predominantly in individuals of European descent. Specifically, GWAS summary statistics for psoriasis were obtained from a European database (NHGRI-EBI GWAS catalog) comprising 15,967 cases and 28,194 controls. This dataset investigated the relationship between psoriasis severity and SNPs. Data for VTE were sourced from another European database (UK Biobank), including 4620 VTE cases and 3,56,574 controls.

### 4.3. Selection and validation of SNPs

We applied 3 criteria to select relevant SNPs for the analysis. Firstly, SNPs associated with psoriasis were chosen based on a genome-wide significance threshold of *P* < 5 × 10^−8^. Secondly, SNPs linked to potential confounders or outcomes were excluded using Linkage Disequilibrium Link (https://ldlink.nih.gov/). Lastly, we assessed the independence of the selected SNPs by examining pairwise-linkage disequilibrium. SNPs with an *r*^2^ > 0.001 (within a clumping window of 10,000 kb) were removed if they were highly correlated with other SNPs or had a higher *P*-value. Prior to conducting the MR analysis, we also performed data harmonization steps to ensure that the effects of SNPs on the exposure and the outcome corresponded to the same allele.

### 4.4. MR analysis

A 2-sample MR approach was employed to estimate the causal effect of genetically predicted psoriasis on VTE in the Mendelian Randomization R package. Cochrane’s *Q*-value was used to assess heterogeneity among the IVs selected for the analysis. Since there was notable heterogeneity among these IVs, an inverse-variance weighted meta-analysis under a random-effects model was considered the primary analysis. Additionally, 2 sensitivity analyses were performed: the weighted-median method and Mendelian randomization–Egger regression. The Mendelian randomization–Egger regression method was specifically used to evaluate potential horizontal pleiotropy of the selected IVs. Furthermore, a leave-one-out sensitivity analysis was conducted to assess the impact of individual SNPs on the overall estimates, ensuring robustness of the findings.

## 5. Results

### 5.1. Observed associations between psoriasis and VTE

**Figure 1.**
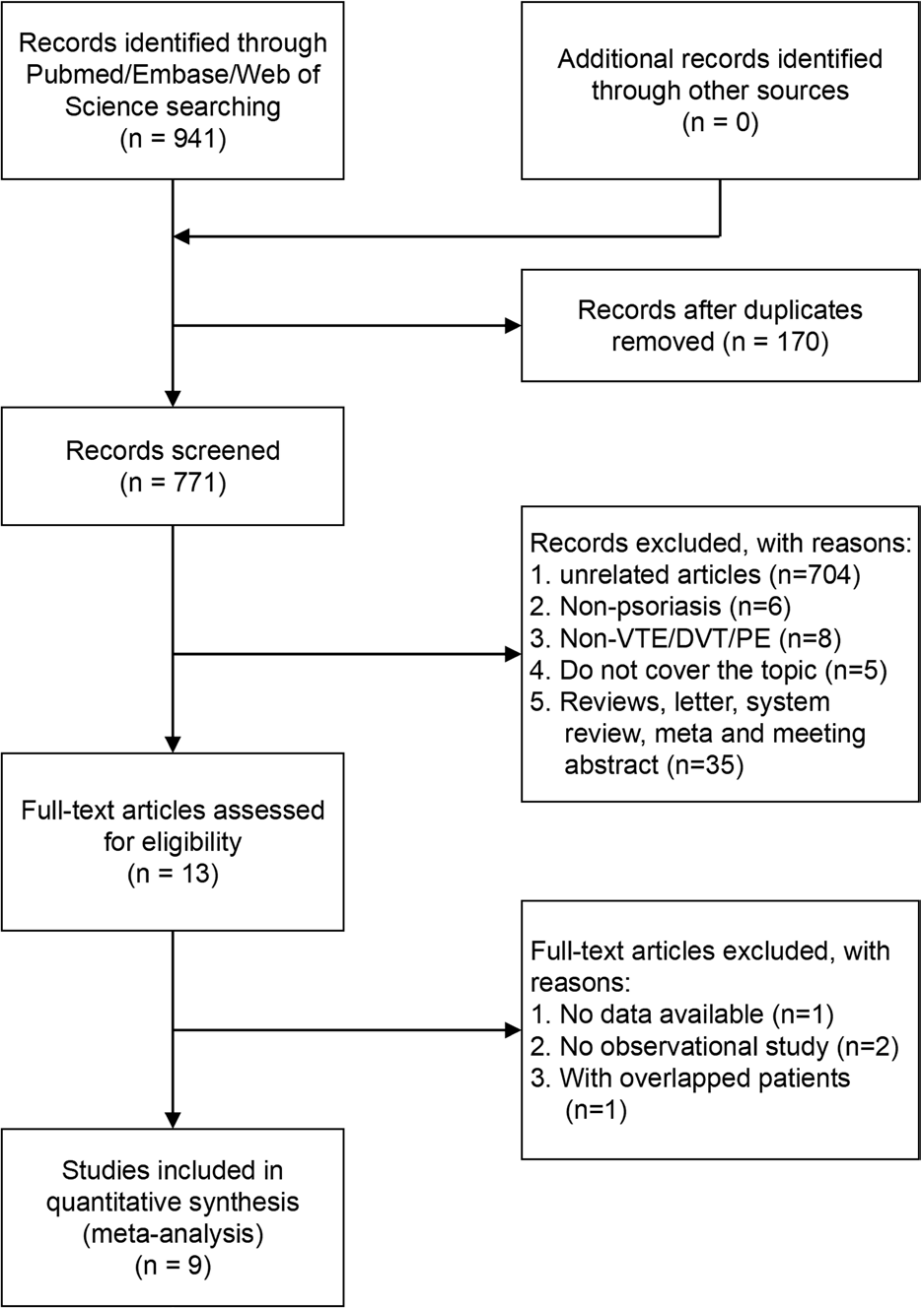
Flow chart of the included studies. DVT = deep vein thrombosis, PE = pulmonary embolism, VTE = venous thromboembolism.

**Figure 2.**
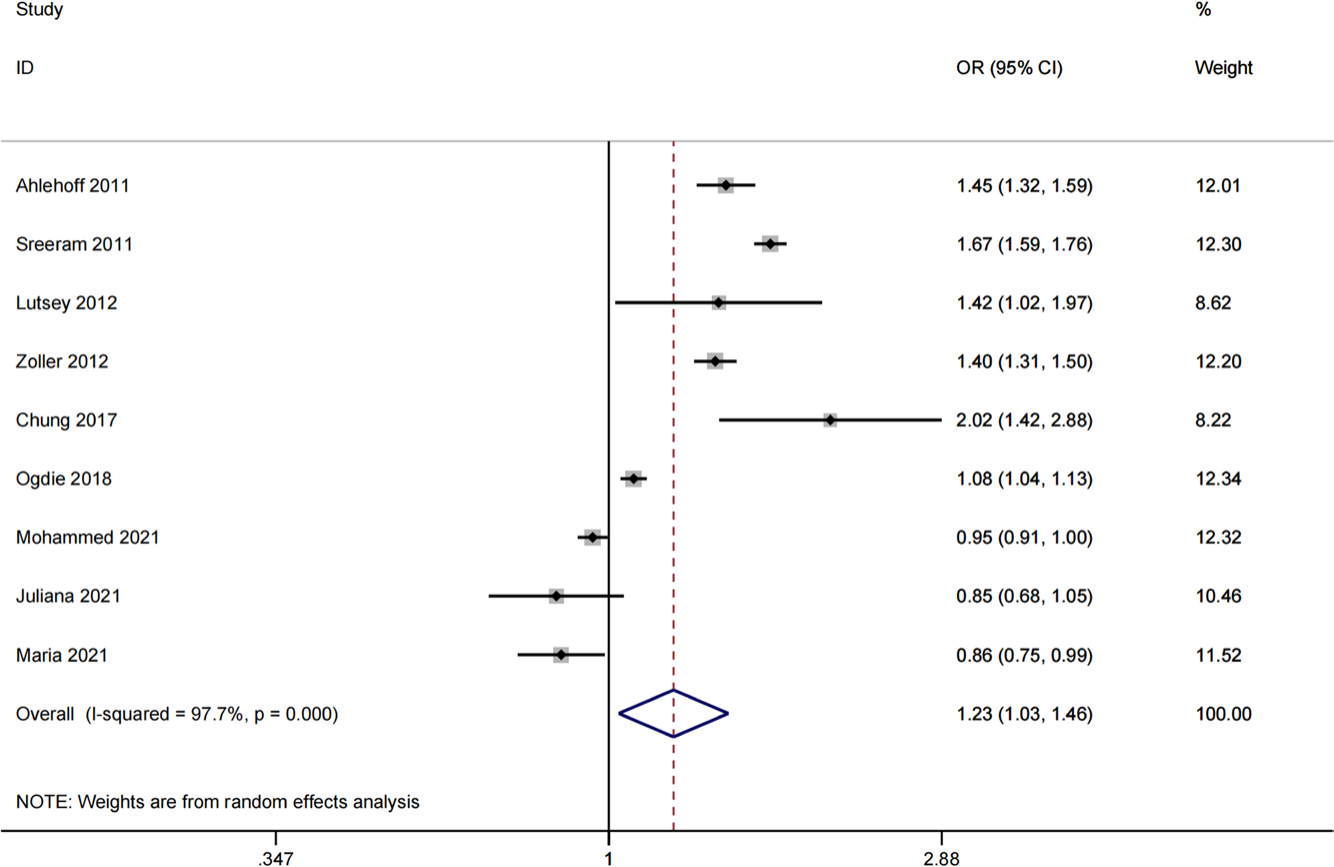
Meta-analysis of the association between psoriasis and VTE. Results are presented as individual and pooled OR, and 95% CI. CI = confidence interval, OR = odds ratio, VTE = venous thromboembolism.

Out of a total of 941 articles identified in the search, 9 studies were included for meta-analysis.^[13–18,24–26]^ The flow chart in Figure 1 summarizes the selection process. The studies encompassed European, North American, and Asian populations. Multivariable analysis provided the HRs/RRs/SIRs/ORs and their corresponding CIs. Table 1 presents the main characteristics of the included studies. We treated HRs, RRs, and SIRs as ORs and performed statistical analysis. Due to significant heterogeneity (*I*^2^ = 97.7%, *P* < .001), a random-effects model was employed. Overall, patients with psoriasis had a higher odd of overall VTE (OR: 1.23, 95% CI: 1.03–1.46), as shown in the forest plot (Fig. 2). Subgroup analyses revealed that psoriasis was associated with an increased incidence of VTE in European (OR: 1.57, 95% CI: 1.34–1.70) and Asian (OR: 2.02, 95% CI: 1.42–2.88) populations, while no significant association was observed in North American studies (OR: 0.98, 95% CI: 0.88–1.10). The RR and OR demonstrated a substantial increase in the risk of VTE among psoriasis patients (RR: 1.30, 95% CI: 1.01–1.68; OR: 0.95, 95% CI: 0.90–0.99), as did the SIR (SIR: 1.40, 95% CI: 1.31–1.50). Conversely, the HR (HR: 1.19, 95% CI: 0.94–1.51) did not show a significant association. Psoriasis was found to increase the incidence of VTE-only (OR: 1.27, 95% CI: 1.03–1.56), but no significant association was observed with PE (OR: 1.13, 95% CI: 0.70–1.83) and DVT (OR: 0.85, 95% CI: 0.65–1.11; Table 2).

**Table 2:** Subgroup analysis of the association between psoriasis and VTE in the meta-analysis.

Analysis	N	References	Random-effects model		Fixed-effects model		Heterogeneity	
			OR (95% CI)	*P*	OR (95% CI)	*P*	*I* ^2^	*P*_h_
Subgroup 1: European	3	13,14,16	1.57 (1.34–1.70)	.00	1.55 (1.49–1.61)	.00	89.2%	0.00
North American	5	15,18,24–26	0.98 (0.88–1.10)	.76	1.01 (0.98–1.04)	.39	86.0%	0.00
Asian	1	17	2.02 (1.42–2.88)	.00	2.02 (1.42–2.88)	.00	–	–
Subgroup 2: RR	3	13,14,25	1.30 (1.01–1.68)	.04	1.57 (1.50–1.64)	.00	94.8%	0.00
HR	4	15,17,18,26	1.19 (0.94–1.51)	.14	1.07 (1.03–1.12)	.00	87.8%	0.00
SIR	1	16	1.40 (1.31–1.50)	.00	1.40 (1.31–1.50)	.00	–	–
OR	1	24	0.95 (0.90–0.99)	.04	0.95 (0.90–0.99)	.04	–	–
Subgroup 3: VTE-only	7	13–15,17,18,24,26	1.27 (1.03–1.56)	.03	1.18 (1.15–1.21)	.00	98.1%	0.00
PE	2	16,25	1.13 (0.70–1.83)	.62	1.38 (1.29–1.47)	.00	94.8%	0.00
DVT	1	25	0.85 (0.65–1.11)	.22	0.85 (0.65–1.11)	.22	–	–

CI = confidence interval, DVT = deep vein thrombosis, HR = hazard ratio, OR = odds ratio, PE = pulmonary embolism, *P*_h_ = *P* for heterogeneity (the corresponding *P*-value of Cochran *Q* test), RR = relative risk, SIR = standardized incidence ratio, VTE = venous thromboembolism.

To assess the robustness of our main result, we performed a sensitivity analysis by systematically excluding individual study analyses. However, the pooled OR remained unchanged (Fig. S2, Supplemental Digital Content, https://links.lww.com/MD/P852). In order to explore potential sources of heterogeneity, we conducted a meta-regression analysis, considering factors such as study region, observation indicator (HRs/RRs/SIRs/ORs), and trait (VTE/DVT/PE). The results of the meta-regression analysis are presented in Table S1, Supplemental Digital Content, https://links.lww.com/MD/P853, and these measures did not fully explain the source of heterogeneity. Subsequently we proceeded to detect publication bias, a funnel plot was generated, and both Begg’s test and Egger’s test were conducted. Although the funnel plot showed some asymmetry (Fig. S3, Supplemental Digital Content, https://links.lww.com/MD/P852), there was no significant publication bias identified in this study (the corresponding *P*-values from Begg’s test and Egger’s test were 0.951 and 0.404, respectively).

### 5.2. Genetic associations between psoriasis and VTE

We examined a total of 85,36,277 SNPs from 44,161 samples. Among those, we identified 59 SNPs associated with psoriasis in Europeans, meeting the significance threshold of *P* < 5 × 10^−8^ and *r*^2^ > 0.001 (Table S2, Supplemental Digital Content, https://links.lww.com/MD/P853). However, 21 of these SNPs were found to have pleiotropic effects on other traits such as type 1/2 diabetes, BMI, smoking, or DVT. To adhere to the assumptions of MR, we excluded these pleiotropic SNPs from our analysis. Therefore, we proceeded with 38 SNPs associated with psoriasis in Europeans as IVs in our MR analyses (Table 3).

**Table 3:** SNPs associated with psoriasis were used as IVs in the MR analyses.

SNP	β	SE	*P*
rs10888503	0.1866	0.0171	1.43615e−27
rs11581607	−0.3647	0.0360	4.55827e−24
rs59960858	−0.2086	0.0243	1.05512e−17
rs12133684	0.1218	0.0206	3.25702e−09
rs6894840	0.1152	0.0166	3.42295e−12
rs11135059	− 0.3078	0.0178	4.40656e−67
rs115059666	0.5147	0.0744	4.46684e−12
rs9468618	−0.1856	0.0300	6.48605e−10
rs9258357	0.2839	0.0233	3.48498e−34
rs9264277	0.3705	0.0180	1.90985e−94
rs2735009	−0.2952	0.0204	1.63192e−47
rs12211087	1.3769	0.0253	1.00000e−200
rs28752856	1.1163	0.0225	1.00000e−200
rs1611236	0.1230	0.0172	7.89587e−13
rs111818167	0.6916	0.0202	1.00000e−200
rs9504361	−0.1068	0.0164	6.44021e−11
rs4712520	0.1301	0.0212	8.27409e−10
rs9277939	0.2280	0.0253	2.16322e−19
rs2451258	−0.0985	0.0169	5.26199e−09
rs11767350	−0.0998	0.0164	1.19300e−09
rs4978343	−0.0988	0.0164	1.76701e−09
rs9695923	−0.0955	0.0165	7.08207e−09
rs1108618	−0.1098	0.0165	3.24788e−11
rs118002009	−0.1020	0.0169	1.61901e−09
rs2066819	−0.3283	0.0348	4.28055e−21
rs9513593	−0.1197	0.0205	5.33495e−09
rs7141014	−0.1123	0.0203	3.07496e−08
rs28510484	−0.1398	0.0231	1.37101e−09
rs2021511	−0.1163	0.0181	1.20301e−10
rs73986523	−0.2122	0.0380	2.41402e−08
rs957970	0.0929	0.0168	3.05401e−08
rs55868394	0.1389	0.0246	1.70801e−08
rs2301368	0.1073	0.0168	1.60901e−10
rs559406	−0.0924	0.0161	1.01899e−08
rs142502677	0.1035	0.0185	2.21998e−08
rs11085744	0.1146	0.0166	5.21315e−12
rs2638281	0.0976	0.0164	2.46297e−09
rs131656	0.1140	0.0202	1.68500e−08

IV = instrumental variables, MR= Mendelian randomization, SE = standard error, SNP = single nucleotide polymorphism.

Due to the presence of strong heterogeneity among these IVs, we utilized a random-effects model for our analysis. Remarkably, we found no evidence to suggest that genetically predicted psoriasis is associated with an increased odd of VTE (inverse-variance weighted OR: 1.000, 95% CI: 0.999–1.001, *P* = .639). Moreover, no evidence of directional pleiotropy was detected. Our leave-one-out sensitivity analysis demonstrated that the overall estimates were not significantly affected by any individual SNP (Fig. S4, Supplemental Digital Content, https://links.lww.com/MD/P852). The scatter plot and forest plot in Figs. S5 and S6, Supplemental Digital Content, https://links.lww.com/MD/P852, respectively, depict similar results regarding the association between psoriasis and VTE.

## 6. Discussion

This study aimed to investigate the association between psoriasis and VTE using both meta-analysis and MR. Previous studies have reported significant variations in the prevalence of psoriasis across different regions. For instance, the incidence rate was 129 cases per 1,00,000 people in the US in 2013,^[29]^ 65 cases per 1,00,000 people in Russia in 2016,^[30]^ 151.2 cases per 1,00,000 people in Denmark in 2012,^[31]^ and 30.3 cases per 1,00,000 people in Taiwan in 2013.^[32]^ Moreover, the risk of VTE among individuals with psoriasis also demonstrated regional variations. The findings from the meta-analysis revealed a positive association between psoriasis and VTE (OR: 1.23, 95% CI: 1.03–1.46). Subgroup analyses further indicated that the odds of VTE associated with psoriasis varied across different regions. Subsequently, we conducted a MR analysis using a European dataset, as there were limited instrumental SNPs available from other psoriasis datasets. Our 2-sample MR analysis, utilizing a European VTE dataset, did not provide evidence of a causal relationship between psoriasis and VTE. The results from the 2 different research methods were not consistent.

Several studies have reported an elevated risk of VTE in patients with psoriasis.^[13–19]^ Chronic inflammatory disorders are known to contribute to a hypercoagulable state and may influence thrombosis.^[12,33]^ Additionally, inflammatory factors such as tumor necrosis factor, interleukins-10, interleukins-6, and interleukins-4 have been shown to increase the incidence of VTE,^[34,35]^ Traditional risk factors like obesity, surgery, and malignancy also exert their effects by activating inflammatory pathways.^[36,37]^ Psoriasis, which is associated with platelet activation, further increases the risk of thrombosis.^[38]^ These mechanistic explanations support the observed correlation. However, there are studies that do not find such an association.^[24–26]^ Some scholars suggest that the overall inflammatory burden in chronic inflammatory skin diseases, such as psoriasis, may be insufficient to increase the incidence of thrombosis.^[26]^ It is important to note that current American guidelines do not consider psoriasis a risk factor for VTE and do not provide specific recommendations for thrombosis prophylaxis in psoriasis patients.^[39]^

Some explanations for the inconsistencies between meta-analysis and MR: Firstly, the meta-analysis conducted in this study is an observational study that combines data from multiple studies, providing a larger sample size and some representativeness. However, it is important to note that there may be inherent heterogeneity and biases in the included studies, which could affect the overall findings. Second, most of the included studies in the meta-analysis adopted a retrospective cohort design, which is particularly prone to selection bias and recall bias. These limitations not only reduce the internal validity but also prevent robust inference regarding temporality and causality. While our meta-analysis primarily assesses associations, its observational nature limits our ability to draw conclusions about the direction or causal nature of the relationship. Thirdly, the studies reported different effect measures, including ORs, RRs, HRs, and SIRs. To enable consistent pooling, we converted all measures to ORs using established methods. While this approach allows for unified effect size estimation, it requires assumptions – such as rare disease incidence and constant baseline hazard – which may not uniformly hold across studies. As such, this transformation might introduce approximation bias and should be interpreted cautiously. Future studies reporting consistent and directly comparable effect measures are needed to further validate these findings. On the other hand, MR, similar to a randomized controlled trial, uses genetic variants as IVs to assess causality, providing a stronger level of evidence. However, the results of MR can still be influenced by factors such as sample size and selection. Secondly, the relationship between psoriasis and VTE may be influenced by individual variations and environmental factors, contributing to the observed discrepancies. The meta-analysis in this study included populations of different races from 3 different regions, while MR primarily focused on European populations. As shown in Figure 2, although the overall effect size suggests a positive correlation between psoriasis and VTE, recent observational studies from the United States did not find a significant association, which is consistent with the MR results. Therefore, it is important to acknowledge that the conclusions drawn from this study may only be applicable to specific populations and conditions, and the generalization to all psoriasis or VTE patients should be done with caution. Finally, it is crucial to note that the inconsistencies observed between the meta-analysis and MR results do not necessarily indicate that 1 is incorrect. Instead, these discrepancies highlight the need for further in-depth research and exploration to gain a comprehensive understanding of the relationship between psoriasis and VTE. Future studies should consider larger sample sizes, more precise study designs, and investigate specific subtypes of psoriasis and VTE to enhance our understanding of this association.

This study serves as an initial investigation into the link between psoriasis and VTE, highlighting the need for further research to verify and elucidate this association. Our goal is to conduct more comprehensive studies, generating robust evidence that can guide clinical practice and treatment approaches. By doing so, we aim to improve the prevention and management of VTE events in individuals with psoriasis.

## Author contributions

**Conceptualization:** Mingzhu Yang, Hao Xu.

**Data curation:** Mingzhu Yang, Hao Xu.

**Formal analysis:** Mingzhu Yang, Haiwei Zhang, Jiao Ma, Chen Xing.

**Investigation:** Mingzhu Yang, Hao Xu.

**Methodology:** Mingzhu Yang, Haiwei Zhang, Jiao Ma, Chen Xing, Hao Xu.

**Project administration:** Hao Xu.

**Software:** Mingzhu Yang, Haiwei Zhang, Jiao Ma, Chen Xing.

**Supervision:** Hao Xu.

**Validation:** Hao Xu.

**Visualization:** Hao Xu.

**Writing – original draft:** Mingzhu Yang.

**Writing – review & editing:** Hao Xu.

## Supplementary Material


