# Supplementary material for: Investigating the relationship between psoriasis and venous thromboembolism using systematic review, meta-analysis and Mendelian randomization study
Source: Medicine (Baltimore). 2025 Sep 5;104(36):e44311. doi: 10.1097/MD.0000000000044311 (PMC12419365; doi:10.1097/MD.0000000000044311)

Supplementary Figure S1. Risk of bias graph of all the included studies.

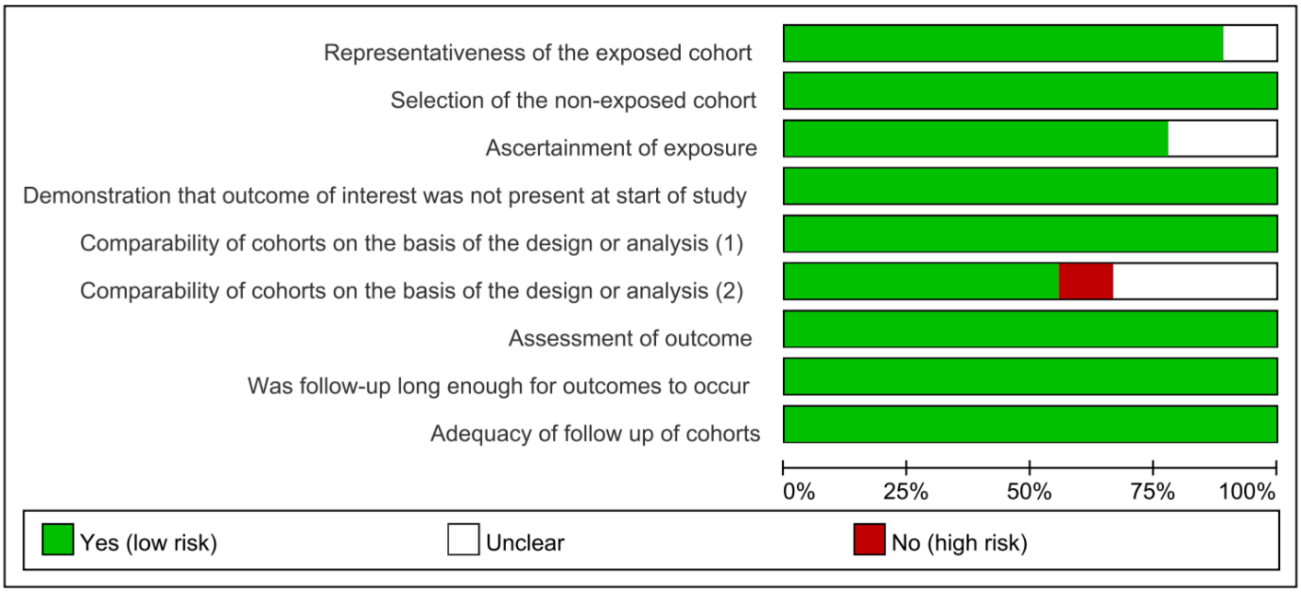

Supplementary Figure S2. Sensitivity analysis in the meta-analysis.

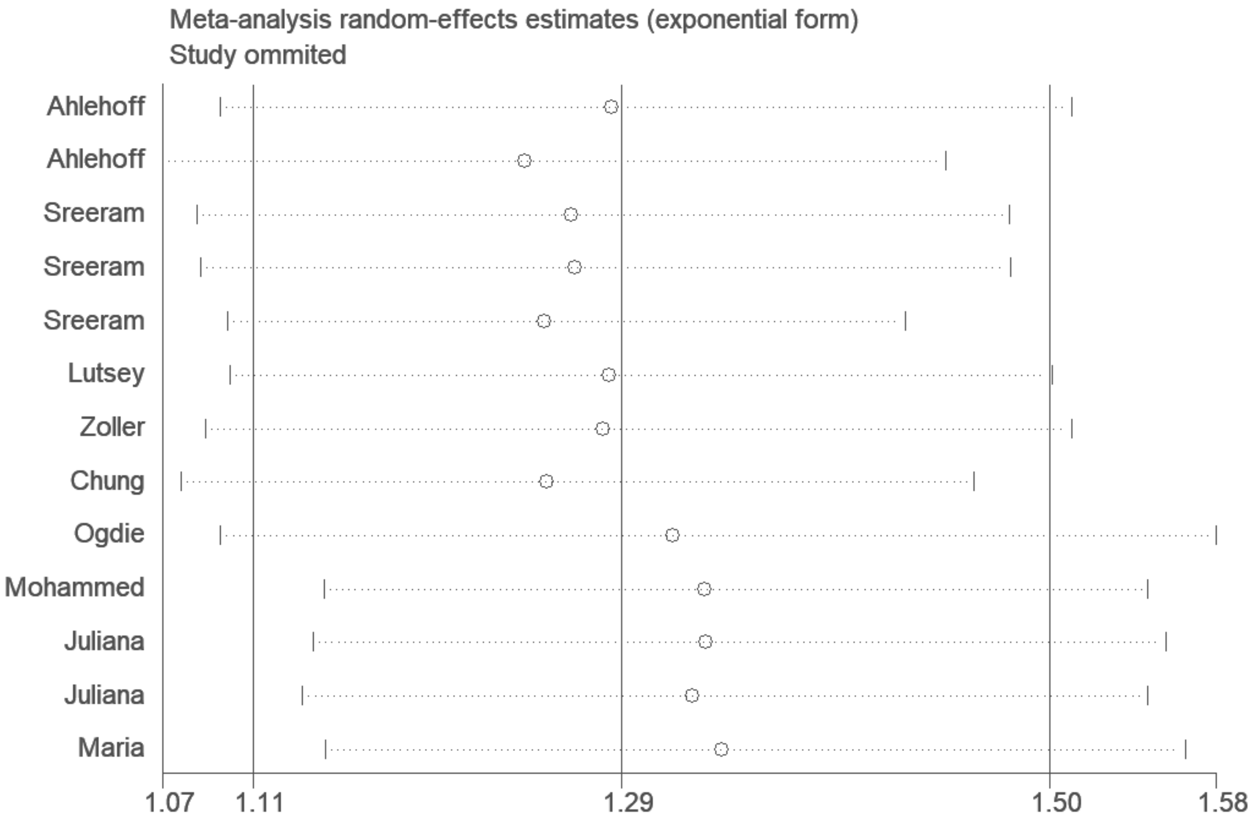

Supplementary Figure S3. The funnel plot of publication bias in the meta-analysis.

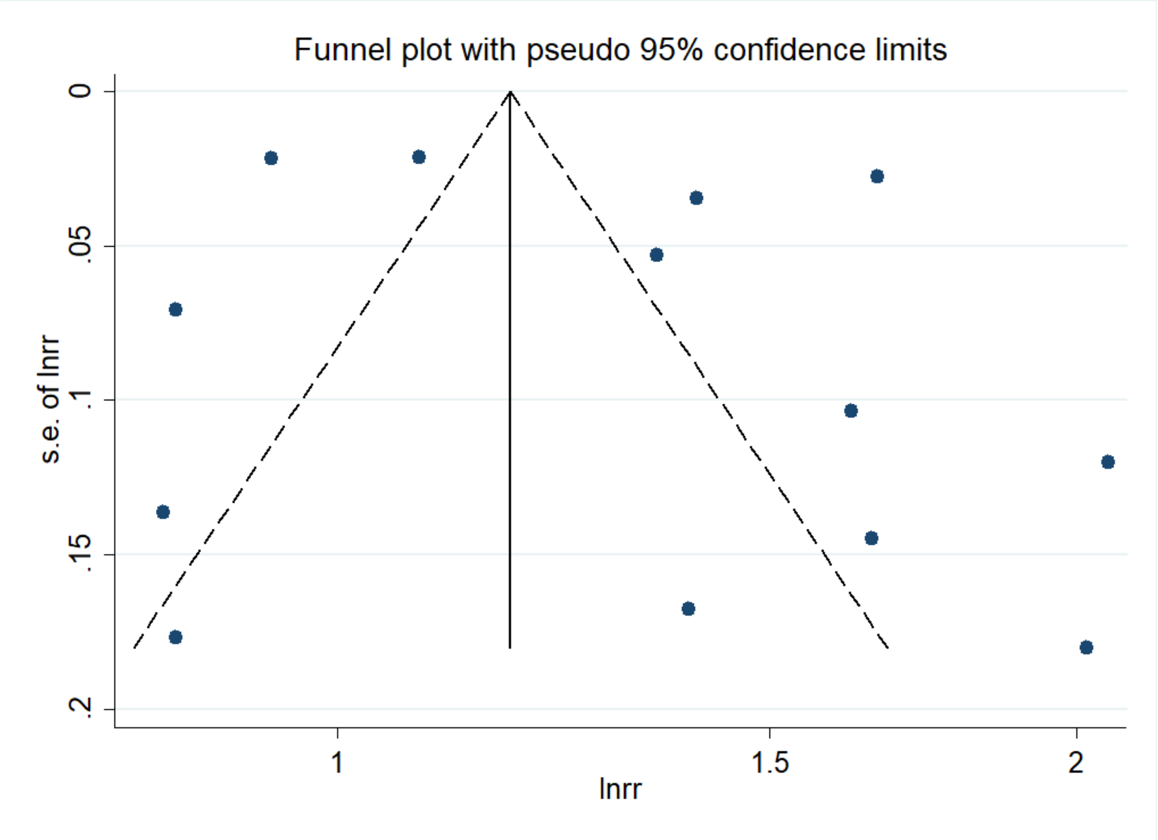

Supplementary Figure S4. Leave-one out sensitivity analysis in the MR.

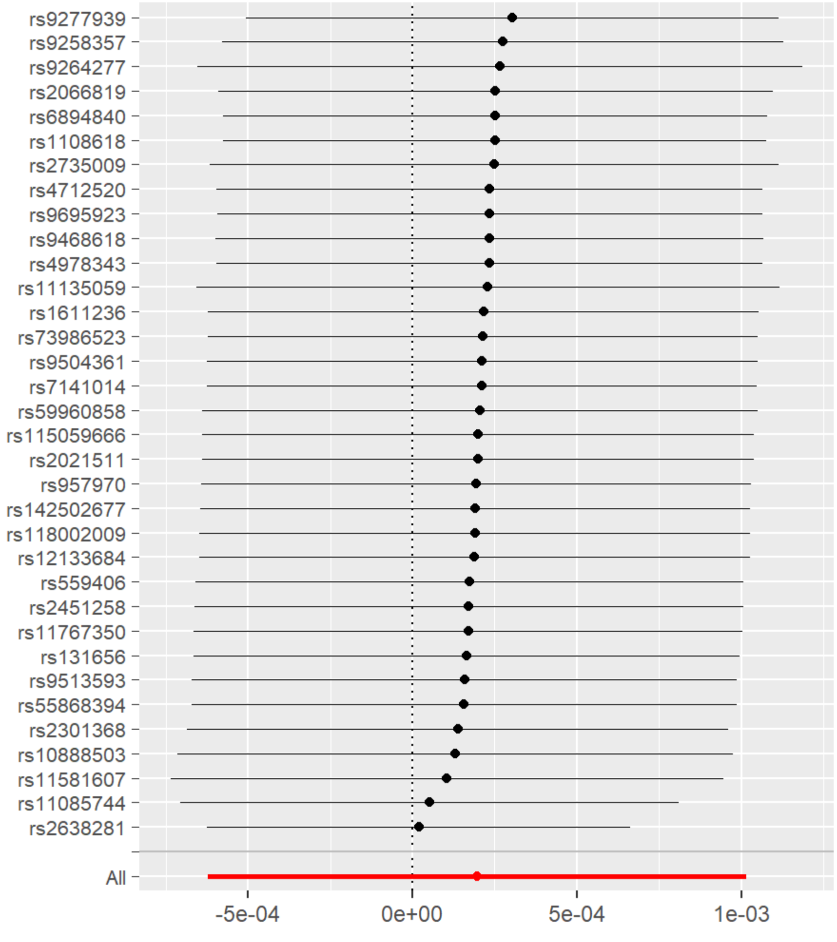

MR leave-one-out sensitivity analysis for psoriasis on VTE

Supplementary Figure S5. Scatter plot using all IVs in the MR.

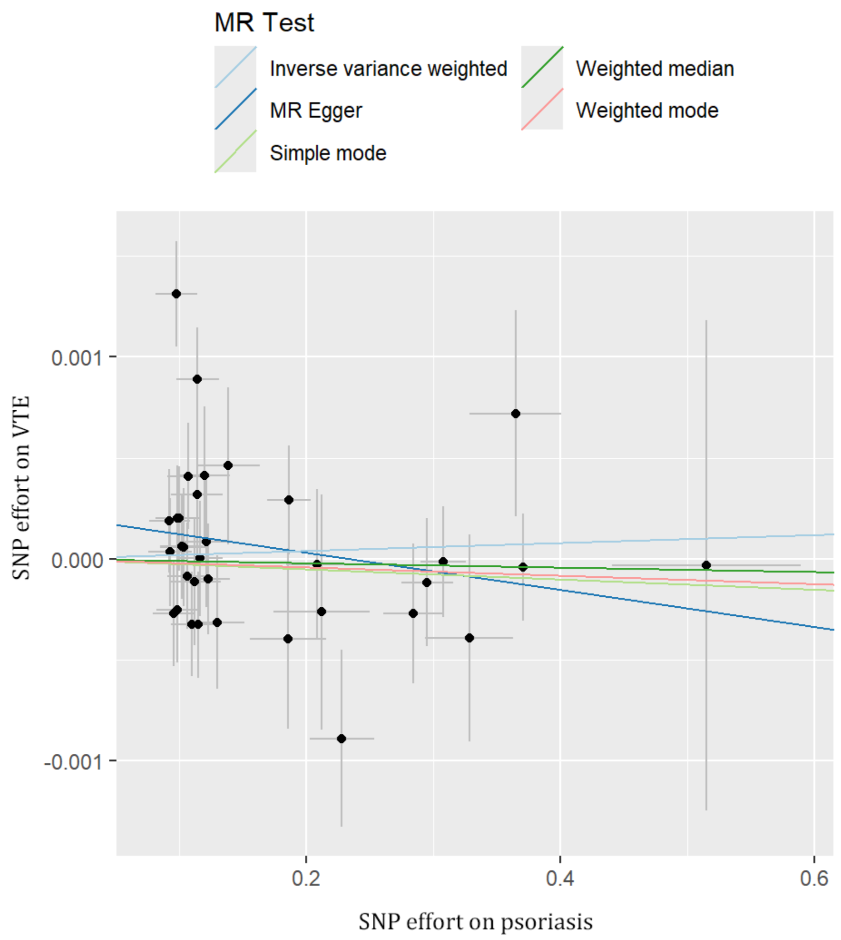

Supplementary Figure S6. Forest plot in the MR.

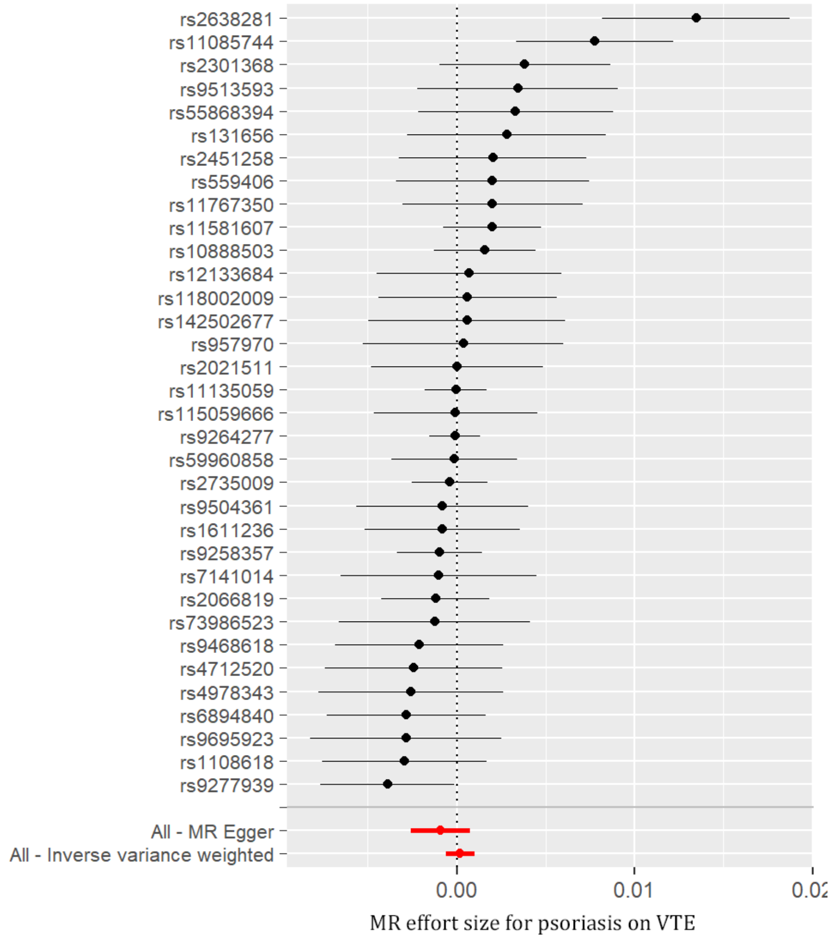

Supplement: Supplementary file 1 [file medi-104-e44311-s001.pdf]
