# Supplementary material for: Investigating the relationship between psoriasis and venous thromboembolism using systematic review, meta-analysis and Mendelian randomization study
Source: Medicine (Baltimore). 2025 Sep 5;104(36):e44311. doi: 10.1097/MD.0000000000044311 (PMC12419365; doi:10.1097/MD.0000000000044311)
Supplement: Supplementary file 2 [file medi-104-e44311-s002.docx]

**Supplementary Table S1. Meta-regression for the potential heterogeneity in the meta-analysis.**

| Covariates | Exp(b) | SE | t | *P*-value | 95% CI | |
| --- | --- | --- | --- | --- | --- | --- |
|  |  |  |  |  | LCI | HCI |
| Study region | -0.202 | 0.138 | -1.48 | 0.168 | -0.506 | 0.100 |
| Observation indicator | -0.098 | 0.092 | -1.06 | 0.312 | -0.300 | 0.105 |
| Trait | -0.226 | 0.140 | -1.61 | 0.137 | -0.535 | 0.084 |

**Supplementary Table S2. SNPs associated with psoriasis of European.**

| SNP | Beta | SE | *P* |
| --- | --- | --- | --- |
| rs10888503 | 0.1866 | 0.0171 | 1.43615e-27 |
| rs438650 | 0.1244 | 0.0195 | 1.76401e-10 |
| rs11581607 | -0.3647 | 0.0360 | 4.55827e-24 |
| rs59960858 | -0.2086 | 0.0243 | 1.05512e-17 |
| rs11249215 | 0.1403 | 0.0160 | 2.14190e-18 |
| rs12133684 | 0.1218 | 0.0206 | 3.25702e-09 |
| rs35741374 | 0.1634 | 0.0163 | 1.07795e-23 |
| rs2111485 | 0.1616 | 0.0166 | 1.75792e-22 |
| rs771576 | 0.1029 | 0.0169 | 1.08101e-09 |
| rs3906814 | -0.1144 | 0.0161 | 1.30407e-12 |
| rs6894840 | 0.1152 | 0.0166 | 3.42295e-12 |
| rs39841 | -0.1624 | 0.0176 | 2.56271e-20 |
| rs1295685 | 0.1759 | 0.0206 | 1.15798e-17 |
| rs11135059 | -0.3078 | 0.0178 | 4.40656e-67 |
| rs115059666 | 0.5147 | 0.0744 | 4.46684e-12 |
| rs12188300 | 0.5005 | 0.0264 | 5.38642e-80 |
| rs9468618 | -0.1856 | 0.0300 | 6.48605e-10 |
| rs9258357 | 0.2839 | 0.0233 | 3.48498e-34 |
| rs9264277 | 0.3705 | 0.0180 | 1.90985e-94 |
| rs2735009 | -0.2952 | 0.0204 | 1.63192e-47 |
| rs12211087 | 1.3769 | 0.0253 | 1.00000e-200 |
| rs28752856 | 1.1163 | 0.0225 | 1.00000e-200 |
| rs1611236 | 0.1230 | 0.0172 | 7.89587e-13 |
| rs111818167 | 0.6916 | 0.0202 | 1.00000e-200 |
| rs582757 | -0.1846 | 0.0176 | 1.15107e-25 |
| rs9504361 | -0.1068 | 0.0164 | 6.44021e-11 |
| rs4712520 | 0.1301 | 0.0212 | 8.27409e-10 |
| rs9277939 | 0.2280 | 0.0253 | 2.16322e-19 |
| rs9481169 | 0.3716 | 0.0263 | 2.47286e-45 |
| rs2451258 | -0.0985 | 0.0169 | 5.26199e-09 |
| rs11767350 | -0.0998 | 0.0164 | 1.19300e-09 |
| rs4978343 | -0.0988 | 0.0164 | 1.76701e-09 |
| rs9695923 | -0.0955 | 0.0165 | 7.08207e-09 |
| rs11795343 | -0.1062 | 0.0164 | 1.02499e-10 |
| rs2675662 | -0.1184 | 0.0167 | 1.48799e-12 |
| rs1108618 | -0.1098 | 0.0165 | 3.24788e-11 |
| rs1648153 | -0.1388 | 0.0164 | 2.93089e-17 |
| rs118002009 | -0.1020 | 0.0169 | 1.61901e-09 |
| rs10893884 | -0.1060 | 0.0160 | 3.98658e-11 |
| rs2066819 | -0.3283 | 0.0348 | 4.28055e-21 |
| rs9591325 | -0.1900 | 0.0324 | 4.49997e-09 |
| rs9513593 | -0.1197 | 0.0205 | 5.33495e-09 |
| rs8016947 | 0.1476 | 0.0162 | 8.17523e-20 |
| rs7141014 | -0.1123 | 0.0203 | 3.07496e-08 |
| rs28510484 | -0.1398 | 0.0231 | 1.37101e-09 |
| rs2021511 | -0.1163 | 0.0181 | 1.20301e-10 |
| rs4889526 | 0.1216 | 0.0165 | 1.62405e-13 |
| rs73986523 | -0.2122 | 0.0380 | 2.41402e-08 |
| rs957970 | 0.0929 | 0.0168 | 3.05401e-08 |
| rs55868394 | 0.1389 | 0.0246 | 1.70801e-08 |
| rs28998802 | 0.2113 | 0.0222 | 1.88799e-21 |
| rs2301368 | 0.1073 | 0.0168 | 1.60901e-10 |
| rs559406 | -0.0924 | 0.0161 | 1.01899e-08 |
| rs142502677 | 0.1035 | 0.0185 | 2.21998e-08 |
| rs11085744 | 0.1146 | 0.0166 | 5.21315e-12 |
| rs2638281 | 0.0976 | 0.0164 | 2.46297e-09 |
| rs34536443 | -0.6799 | 0.0504 | 2.01790e-41 |
| rs6063454 | -0.1551 | 0.0166 | 1.07696e-20 |
| rs131656 | 0.1140 | 0.0202 | 1.68500e-08 |
